# 2,2-Diethyl 4-methyl 5-(4-nitro­phen­yl)-4-phenyl­pyrrolidine-2,2,4-tricarboxyl­ate

**DOI:** 10.1107/S1600536811036038

**Published:** 2011-09-14

**Authors:** Long He

**Affiliations:** a College of Chemistry and Chemical Engineering, China West Normal University, Nanchong 637002, People’s Republic of China

## Abstract

The title compound, C_24_H_26_N_2_O_8_, was synthesized by the cyclo­addition reaction of methyl 2-phenyl­acrylate, diethyl 2-amino­malonate and 4-nitro­benzaldehyde. The pyrrolidine ring exhibits an envelope conformation. The two benzene rings are located on opposite sides of the pyrrolidine ring and subtend a dihedral angle of 59.16 (14)°. The crystal packing is stabilized by N—H⋯O and weak C—H⋯O hydrogen bonding.

## Related literature

For the biological activity of pyrrolidine derivatives, see: Coldham & Hufton (2005 ▶); Nair & Suja (2007 ▶); Pandey *et al.* (2006 ▶); Sardina & Rapoport (1996 ▶). For a related structure, see: Yu *et al.* (2007 ▶).
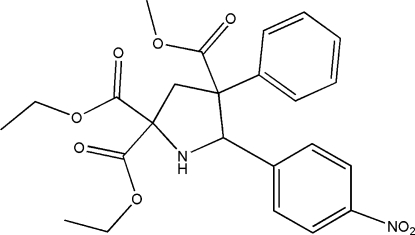


## Experimental

### 

#### Crystal data

C_24_H_26_N_2_O_8_
*M* *_r_* = 470.47
Orthorhombic, 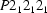

*a* = 9.7948 (1) Å
*b* = 10.9356 (2) Å
*c* = 22.3240 (3) Å
*V* = 2391.17 (6) Å^3^
*Z* = 4
Cu *K*α radiation
μ = 0.83 mm^−1^
*T* = 120 K
0.44 × 0.40 × 0.36 mm

#### Data collection

Gemini S Ultra, Oxford Diffraction diffractometer
Absorption correction: multi-scan (*CrysAlis PRO*; Oxford Diffraction, 2009 ▶) *T*_min_ = 0.712, *T*_max_ = 0.755
21308 measured reflections
4686 independent reflections
4613 reflections with *I* > 2σ(*I*)
*R*_int_ = 0.018

#### Refinement

*R*[*F* ^2^ > 2σ(*F* ^2^)] = 0.025
*wR*(*F* ^2^) = 0.068
*S* = 1.02
4686 reflections
314 parameters
1 restraint
H atoms treated by a mixture of independent and constrained refinement
Δρ_max_ = 0.18 e Å^−3^
Δρ_min_ = −0.19 e Å^−3^
Absolute structure: Flack (1983 ▶), 1995 Friedel pairs
Flack parameter: 0.05 (10)

### 

Data collection: *CrysAlis PRO* (Oxford Diffraction, 2009 ▶); cell refinement: *CrysAlis PRO*; data reduction: *CrysAlis PRO*; program(s) used to solve structure: *SHELXS97* (Sheldrick, 2008 ▶); program(s) used to refine structure: *SHELXL97* (Sheldrick, 2008 ▶); molecular graphics: *ORTEP-3* (Farrugia, 1997 ▶); software used to prepare material for publication: *SHELXL97*.

## Supplementary Material

Crystal structure: contains datablock(s) global, I. DOI: 10.1107/S1600536811036038/xu5319sup1.cif

Structure factors: contains datablock(s) I. DOI: 10.1107/S1600536811036038/xu5319Isup2.hkl

Supplementary material file. DOI: 10.1107/S1600536811036038/xu5319Isup3.cml

Additional supplementary materials:  crystallographic information; 3D view; checkCIF report

## Figures and Tables

**Table 1:** Hydrogen-bond geometry (Å, °)

*D*—H⋯*A*	*D*—H	H⋯*A*	*D*⋯*A*	*D*—H⋯*A*
N2—H1⋯O7^i^	0.88 (1)	2.59 (1)	3.360 (4)	148 (1)
C12—H12*C*⋯O1^ii^	0.96	2.58	3.360 (2)	139 (1)

Symmetry codes: (i) 
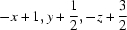
; (ii) 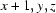.

**Fig. 1.**
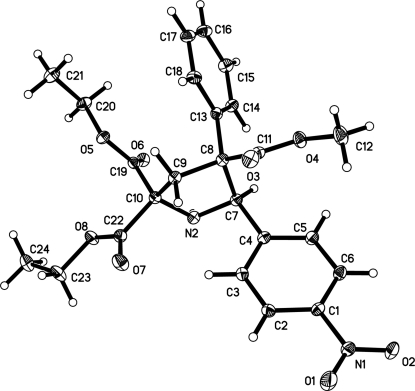
The molecular structure of (I) with 30% probability displacement ellipsoids (arbitrary spheres for H atoms).

## supplementary crystallographic information

### Comment

Substituted pyrrolidine compound is an important class of heterocyclic compounds with wide spread applications to the synthesis of biologically active compounds and natural products (Coldham & Hufton, 2005; Nair & Suja, 2007; Pandey *et al.*, 2006; Sardina & Rapoport, 1996). Its crystal structure is reported here.

The molecular structure of (I) is shown in (Table 2). Bond lengths and angles in (I) are normal. The pyrrolidine ring possesses an envelope conformation. The dihedral angle between the C1—C6 and C13—C18 benzene planes is 59.16 (14)°. The crystal packing is stabilized by N—H···O and C—H···O hydrogen bonds (Table 1).

### Experimental

Diethyl 2-aminomalonate (0.0175 g, 0.1 mmol) were added to a solution of 4-nitrobenzaldehyde (0.018 g, 0.12 mmol) and methyl 2-phenylacrylate (0.08 g, 0.5 mmol) in dichloromethane (1 ml). To the stirred mixture, acetic acid (0.003 g, 0.05 mmol) was added. After the mixture had been stirred at 298 K for 24 h, the reaction was quenched with a saturated solution of sodium bicarbonate (5 ml). The mixture was extracted with diethyl ether, evaporated and separated by flash chromatograghy. A colourless powder was obtained. Single crystals suitable for X-ray diffraction were obtained by slow evaporation of an ethanol solution.

### Refinement

Imino H atom was placed in chemical sensible position and refined isotropically. The remaining carbon-bound H atoms were placed in calculated positions, with C—H = 0.93–0.99 Å, and refined using a riding model, with *U*_iso_(H) = 1.5*U*_eq_(C) for methyl H atoms and 1.2*U*_eq_(C) for the others.

### Crystal data

**Table:**

C_24_H_26_N_2_O_8_	*D*_x_ = 1.307 Mg m^−^^3^
*M**_r_* = 470.47	Cu *K*α radiation, λ = 1.54184 Å
Orthorhombic, *P*2_1_2_1_2_1_	Cell parameters from 18450 reflections
*a* = 9.7948 (1) Å	θ = 2.0–72.2°
*b* = 10.9356 (2) Å	µ = 0.83 mm^−^^1^
*c* = 22.3240 (3) Å	*T* = 120 K
*V* = 2391.17 (6) Å^3^	Block, colorless
*Z* = 4	0.44 × 0.40 × 0.36 mm
*F*(000) = 992	

### Data collection

**Table:**

Gemini S Ultra, Oxford Diffraction diffractometer	4686 independent reflections
Radiation source: Enhance Ultra (Cu) X-ray Source	4613 reflections with *I* > 2σ(*I*)
mirror	*R*_int_ = 0.018
Detector resolution: 15.9149 pixels mm^-1^	θ_max_ = 72.3°, θ_min_ = 4.5°
ω scans	*h* = −9→12
Absorption correction: multi-scan (*CrysAlis PRO*; Oxford Diffraction, 2009)	*k* = −13→13
*T*_min_ = 0.712, *T*_max_ = 0.755	*l* = −25→27
21308 measured reflections	

### Refinement

**Table:**

Refinement on *F*^2^	Secondary atom site location: difference Fourier map
Least-squares matrix: full	Hydrogen site location: inferred from neighbouring sites
*R*[*F*^2^ > 2σ(*F*^2^)] = 0.025	H atoms treated by a mixture of independent and constrained refinement
*wR*(*F*^2^) = 0.068	*w* = 1/[σ^2^(*F*_o_^2^) + (0.0432*P*)^2^ + 0.3419*P*] where *P* = (*F*_o_^2^ + 2*F*_c_^2^)/3
*S* = 1.02	(Δ/σ)_max_ = 0.001
4686 reflections	Δρ_max_ = 0.18 e Å^−^^3^
314 parameters	Δρ_min_ = −0.19 e Å^−^^3^
1 restraint	Absolute structure: Flack (1983), 1995 Friedel pairs
Primary atom site location: structure-invariant direct methods	Flack parameter: 0.05 (10)

### Special details

**Table:**

Geometry. All e.s.d.'s (except the e.s.d. in the dihedral angle between two l.s. planes) are estimated using the full covariance matrix. The cell e.s.d.'s are taken into account individually in the estimation of e.s.d.'s in distances, angles and torsion angles; correlations between e.s.d.'s in cell parameters are only used when they are defined by crystal symmetry. An approximate (isotropic) treatment of cell e.s.d.'s is used for estimating e.s.d.'s involving l.s. planes.
Refinement. Refinement of *F*^2^ against ALL reflections. The weighted *R*-factor *wR* and goodness of fit *S* are based on *F*^2^, conventional *R*-factors *R* are based on *F*, with *F* set to zero for negative *F*^2^. The threshold expression of *F*^2^ > σ(*F*^2^) is used only for calculating *R*-factors(gt) *etc*. and is not relevant to the choice of reflections for refinement. *R*-factors based on *F*^2^ are statistically about twice as large as those based on *F*, and *R*- factors based on ALL data will be even larger.

### Fractional atomic coordinates and isotropic or equivalent isotropic displacement parameters (Å^2^)

**Table:**

	*x*	*y*	*z*	*U*_iso_*/*U*_eq_	
O5	0.85432 (8)	0.19604 (7)	0.82698 (3)	0.02452 (17)	
O8	0.54703 (9)	0.22728 (8)	0.84562 (4)	0.02882 (18)	
O6	0.78904 (8)	0.37692 (7)	0.78836 (4)	0.02724 (18)	
O4	0.84169 (9)	0.10400 (8)	0.56346 (4)	0.03091 (19)	
O3	0.78250 (9)	−0.05809 (7)	0.61949 (4)	0.03171 (19)	
O7	0.48509 (10)	0.06449 (9)	0.79096 (4)	0.0385 (2)	
N2	0.60890 (10)	0.26899 (9)	0.70963 (4)	0.02279 (19)	
O2	0.24732 (10)	0.03877 (8)	0.44941 (4)	0.0337 (2)	
O1	0.17733 (13)	−0.06248 (12)	0.52573 (5)	0.0574 (3)	
C9	0.73152 (11)	0.08472 (10)	0.72218 (5)	0.0220 (2)	
H9A	0.6621	0.0239	0.7137	0.026*	
H9B	0.8053	0.0467	0.7444	0.026*	
N1	0.25329 (10)	0.01249 (9)	0.50266 (4)	0.0269 (2)	
C7	0.66436 (11)	0.23550 (10)	0.65042 (5)	0.0215 (2)	
H7	0.7006	0.3086	0.6306	0.026*	
C1	0.35612 (11)	0.07188 (10)	0.54019 (5)	0.0231 (2)	
C6	0.45396 (13)	0.14517 (11)	0.51335 (5)	0.0273 (2)	
H6	0.4533	0.1589	0.4722	0.033*	
C19	0.77782 (11)	0.26768 (10)	0.79176 (5)	0.0221 (2)	
C13	0.92478 (11)	0.20396 (10)	0.67511 (5)	0.0215 (2)	
C23	0.43853 (15)	0.20090 (13)	0.88861 (6)	0.0373 (3)	
H23B	0.4401	0.1151	0.8997	0.045*	
H23A	0.3501	0.2194	0.8713	0.045*	
C10	0.67169 (11)	0.19350 (10)	0.75643 (5)	0.0217 (2)	
C8	0.78413 (11)	0.14446 (9)	0.66437 (5)	0.0212 (2)	
C18	1.02741 (12)	0.13124 (10)	0.70024 (5)	0.0263 (2)	
H18	1.0087	0.0500	0.7094	0.032*	
C2	0.35244 (12)	0.05137 (11)	0.60140 (5)	0.0252 (2)	
H2	0.2852	0.0022	0.6183	0.030*	
C24	0.46374 (15)	0.27937 (13)	0.94241 (6)	0.0378 (3)	
H24A	0.4616	0.3639	0.9309	0.057*	
H24C	0.5516	0.2603	0.9590	0.057*	
H24B	0.3943	0.2643	0.9718	0.057*	
C20	0.96208 (13)	0.25973 (11)	0.85989 (6)	0.0296 (3)	
H20B	0.9225	0.3179	0.8877	0.036*	
H20A	1.0208	0.3037	0.8323	0.036*	
C3	0.45103 (12)	0.10576 (11)	0.63675 (5)	0.0243 (2)	
H3	0.4492	0.0939	0.6780	0.029*	
C4	0.55327 (11)	0.17816 (10)	0.61151 (5)	0.0223 (2)	
C14	0.95634 (12)	0.32441 (10)	0.66059 (5)	0.0254 (2)	
H14	0.8901	0.3744	0.6436	0.030*	
C17	1.15604 (12)	0.17777 (11)	0.71167 (6)	0.0288 (2)	
H17	1.2222	0.1284	0.7292	0.035*	
C16	1.18663 (12)	0.29794 (11)	0.69709 (6)	0.0303 (3)	
H16	1.2732	0.3294	0.7045	0.036*	
C11	0.80173 (11)	0.05013 (10)	0.61454 (5)	0.0240 (2)	
C15	1.08674 (13)	0.37060 (11)	0.67138 (6)	0.0302 (3)	
H15	1.1068	0.4511	0.6612	0.036*	
C22	0.55725 (12)	0.15160 (10)	0.79941 (5)	0.0248 (2)	
C5	0.55285 (12)	0.19731 (11)	0.54968 (5)	0.0271 (2)	
H5	0.6202	0.2460	0.5325	0.033*	
C12	0.84991 (17)	0.02457 (14)	0.51173 (6)	0.0415 (3)	
H12B	0.8772	0.0713	0.4774	0.062*	
H12A	0.7622	−0.0116	0.5044	0.062*	
H12C	0.9158	−0.0387	0.5191	0.062*	
C21	1.04251 (14)	0.16528 (12)	0.89319 (6)	0.0354 (3)	
H21A	1.0860	0.1113	0.8651	0.053*	
H21B	0.9824	0.1191	0.9185	0.053*	
H21C	1.1107	0.2048	0.9173	0.053*	
H1	0.6194 (16)	0.3476 (12)	0.7157 (7)	0.036 (4)*	

### Atomic displacement parameters (Å^2^)

**Table:**

	*U*^11^	*U*^22^	*U*^33^	*U*^12^	*U*^13^	*U*^23^
O5	0.0248 (4)	0.0227 (4)	0.0261 (4)	−0.0011 (3)	−0.0056 (3)	−0.0024 (3)
O8	0.0301 (4)	0.0284 (4)	0.0279 (4)	−0.0067 (3)	0.0083 (3)	−0.0035 (3)
O6	0.0313 (4)	0.0217 (4)	0.0287 (4)	−0.0023 (3)	−0.0017 (3)	−0.0022 (3)
O4	0.0376 (4)	0.0301 (4)	0.0250 (4)	−0.0048 (4)	0.0040 (3)	−0.0057 (3)
O3	0.0395 (5)	0.0214 (4)	0.0342 (4)	−0.0001 (3)	−0.0028 (4)	−0.0042 (3)
O7	0.0411 (5)	0.0389 (5)	0.0354 (5)	−0.0188 (4)	0.0072 (4)	−0.0086 (4)
N2	0.0255 (5)	0.0190 (4)	0.0238 (5)	0.0015 (4)	−0.0022 (4)	−0.0018 (4)
O2	0.0407 (5)	0.0350 (4)	0.0254 (4)	−0.0050 (4)	−0.0103 (4)	0.0014 (3)
O1	0.0617 (7)	0.0747 (8)	0.0358 (5)	−0.0457 (6)	−0.0091 (5)	0.0069 (5)
C9	0.0243 (5)	0.0181 (5)	0.0236 (5)	−0.0012 (4)	−0.0021 (4)	0.0000 (4)
N1	0.0276 (5)	0.0263 (5)	0.0269 (5)	−0.0025 (4)	−0.0035 (4)	−0.0019 (4)
C7	0.0221 (5)	0.0196 (5)	0.0227 (5)	−0.0004 (4)	−0.0016 (4)	0.0014 (4)
C1	0.0234 (5)	0.0212 (5)	0.0246 (5)	0.0003 (4)	−0.0044 (4)	−0.0022 (4)
C6	0.0311 (6)	0.0268 (6)	0.0240 (5)	−0.0031 (5)	−0.0043 (4)	0.0040 (4)
C19	0.0216 (5)	0.0235 (5)	0.0212 (5)	−0.0008 (4)	0.0017 (4)	−0.0019 (4)
C13	0.0227 (5)	0.0218 (5)	0.0200 (5)	−0.0005 (4)	0.0004 (4)	−0.0036 (4)
C23	0.0384 (7)	0.0380 (7)	0.0356 (7)	−0.0109 (6)	0.0165 (6)	−0.0030 (6)
C10	0.0225 (5)	0.0203 (5)	0.0224 (5)	−0.0013 (4)	−0.0007 (4)	0.0004 (4)
C8	0.0230 (5)	0.0178 (5)	0.0229 (5)	−0.0004 (4)	−0.0016 (4)	−0.0003 (4)
C18	0.0254 (5)	0.0221 (5)	0.0315 (6)	0.0022 (4)	0.0013 (5)	−0.0016 (5)
C2	0.0236 (5)	0.0263 (5)	0.0256 (5)	−0.0035 (4)	0.0014 (4)	0.0005 (4)
C24	0.0470 (8)	0.0350 (7)	0.0315 (6)	−0.0039 (6)	0.0122 (6)	−0.0009 (5)
C20	0.0278 (6)	0.0284 (6)	0.0326 (6)	−0.0018 (5)	−0.0076 (5)	−0.0083 (5)
C3	0.0255 (5)	0.0266 (5)	0.0209 (5)	−0.0004 (5)	0.0009 (4)	−0.0003 (4)
C4	0.0223 (5)	0.0194 (5)	0.0252 (5)	0.0010 (4)	−0.0026 (4)	0.0000 (4)
C14	0.0257 (5)	0.0236 (5)	0.0269 (5)	−0.0006 (4)	−0.0038 (4)	0.0003 (4)
C17	0.0230 (5)	0.0302 (6)	0.0332 (6)	0.0059 (5)	−0.0023 (5)	−0.0040 (5)
C16	0.0224 (5)	0.0311 (6)	0.0373 (6)	−0.0015 (5)	−0.0019 (5)	−0.0079 (5)
C11	0.0214 (5)	0.0243 (5)	0.0262 (5)	0.0002 (4)	−0.0034 (4)	−0.0029 (5)
C15	0.0306 (6)	0.0235 (5)	0.0366 (7)	−0.0052 (5)	−0.0019 (5)	−0.0002 (5)
C22	0.0255 (5)	0.0240 (5)	0.0247 (5)	−0.0023 (4)	−0.0009 (4)	0.0003 (4)
C5	0.0288 (5)	0.0264 (6)	0.0263 (5)	−0.0056 (5)	−0.0026 (5)	0.0051 (5)
C12	0.0518 (8)	0.0441 (8)	0.0286 (6)	−0.0079 (6)	0.0080 (6)	−0.0128 (6)
C21	0.0335 (6)	0.0354 (6)	0.0374 (7)	−0.0006 (5)	−0.0115 (5)	−0.0020 (5)

### Geometric parameters (Å, °)

**Table:**

O5—C19	1.3392 (14)	C23—H23B	0.9700
O5—C20	1.4626 (13)	C23—H23A	0.9700
O8—C22	1.3263 (14)	C10—C22	1.5450 (15)
O8—C23	1.4608 (14)	C8—C11	1.5269 (15)
O6—C19	1.2021 (14)	C18—C17	1.3825 (17)
O4—C11	1.3417 (14)	C18—H18	0.9300
O4—C12	1.4473 (15)	C2—C3	1.3816 (16)
O3—C11	1.2034 (14)	C2—H2	0.9300
O7—C22	1.2010 (14)	C24—H24A	0.9600
N2—C10	1.4666 (14)	C24—H24C	0.9600
N2—C7	1.4754 (14)	C24—H24B	0.9600
N2—H1	0.877 (13)	C20—C21	1.4967 (17)
O2—N1	1.2244 (13)	C20—H20B	0.9700
O1—N1	1.2211 (15)	C20—H20A	0.9700
C9—C10	1.5307 (15)	C3—C4	1.3954 (16)
C9—C8	1.5356 (15)	C3—H3	0.9300
C9—H9A	0.9700	C4—C5	1.3961 (16)
C9—H9B	0.9700	C14—C15	1.3945 (17)
N1—C1	1.4622 (14)	C14—H14	0.9300
C7—C4	1.5270 (14)	C17—C16	1.3866 (18)
C7—C8	1.5698 (14)	C17—H17	0.9300
C7—H7	0.9800	C16—C15	1.3849 (18)
C1—C2	1.3852 (16)	C16—H16	0.9300
C1—C6	1.3856 (16)	C15—H15	0.9300
C6—C5	1.3861 (16)	C5—H5	0.9300
C6—H6	0.9300	C12—H12B	0.9600
C19—C10	1.5365 (15)	C12—H12A	0.9600
C13—C14	1.3912 (16)	C12—H12C	0.9600
C13—C18	1.3991 (16)	C21—H21A	0.9600
C13—C8	1.5424 (15)	C21—H21B	0.9600
C23—C24	1.4966 (18)	C21—H21C	0.9600
			
C19—O5—C20	114.85 (9)	C3—C2—C1	118.39 (10)
C22—O8—C23	116.28 (9)	C3—C2—H2	120.8
C11—O4—C12	115.52 (10)	C1—C2—H2	120.8
C10—N2—C7	110.13 (8)	C23—C24—H24A	109.5
C10—N2—H1	113.2 (10)	C23—C24—H24C	109.5
C7—N2—H1	109.8 (10)	H24A—C24—H24C	109.5
C10—C9—C8	102.57 (8)	C23—C24—H24B	109.5
C10—C9—H9A	111.3	H24A—C24—H24B	109.5
C8—C9—H9A	111.3	H24C—C24—H24B	109.5
C10—C9—H9B	111.3	O5—C20—C21	107.50 (10)
C8—C9—H9B	111.3	O5—C20—H20B	110.2
H9A—C9—H9B	109.2	C21—C20—H20B	110.2
O1—N1—O2	122.54 (10)	O5—C20—H20A	110.2
O1—N1—C1	118.44 (10)	C21—C20—H20A	110.2
O2—N1—C1	119.01 (10)	H20B—C20—H20A	108.5
N2—C7—C4	110.44 (9)	C2—C3—C4	121.01 (10)
N2—C7—C8	104.76 (8)	C2—C3—H3	119.5
C4—C7—C8	112.64 (9)	C4—C3—H3	119.5
N2—C7—H7	109.6	C3—C4—C5	118.83 (10)
C4—C7—H7	109.6	C3—C4—C7	120.98 (10)
C8—C7—H7	109.6	C5—C4—C7	120.19 (10)
C2—C1—C6	122.57 (10)	C13—C14—C15	120.41 (11)
C2—C1—N1	118.39 (10)	C13—C14—H14	119.8
C6—C1—N1	119.05 (10)	C15—C14—H14	119.8
C1—C6—C5	117.92 (10)	C18—C17—C16	120.16 (11)
C1—C6—H6	121.0	C18—C17—H17	119.9
C5—C6—H6	121.0	C16—C17—H17	119.9
O6—C19—O5	124.56 (10)	C15—C16—C17	119.23 (11)
O6—C19—C10	123.65 (10)	C15—C16—H16	120.4
O5—C19—C10	111.79 (9)	C17—C16—H16	120.4
C14—C13—C18	118.16 (10)	O3—C11—O4	123.75 (10)
C14—C13—C8	124.17 (10)	O3—C11—C8	125.43 (10)
C18—C13—C8	117.67 (10)	O4—C11—C8	110.82 (9)
O8—C23—C24	107.10 (10)	C16—C15—C14	120.72 (11)
O8—C23—H23B	110.3	C16—C15—H15	119.6
C24—C23—H23B	110.3	C14—C15—H15	119.6
O8—C23—H23A	110.3	O7—C22—O8	124.94 (11)
C24—C23—H23A	110.3	O7—C22—C10	124.38 (10)
H23B—C23—H23A	108.5	O8—C22—C10	110.65 (9)
N2—C10—C9	104.02 (8)	C6—C5—C4	121.25 (11)
N2—C10—C19	110.62 (9)	C6—C5—H5	119.4
C9—C10—C19	114.06 (9)	C4—C5—H5	119.4
N2—C10—C22	107.76 (9)	O4—C12—H12B	109.5
C9—C10—C22	110.94 (9)	O4—C12—H12A	109.5
C19—C10—C22	109.18 (9)	H12B—C12—H12A	109.5
C11—C8—C9	111.27 (9)	O4—C12—H12C	109.5
C11—C8—C13	107.31 (8)	H12B—C12—H12C	109.5
C9—C8—C13	110.39 (9)	H12A—C12—H12C	109.5
C11—C8—C7	111.61 (9)	C20—C21—H21A	109.5
C9—C8—C7	100.70 (8)	C20—C21—H21B	109.5
C13—C8—C7	115.52 (9)	H21A—C21—H21B	109.5
C17—C18—C13	121.30 (11)	C20—C21—H21C	109.5
C17—C18—H18	119.3	H21A—C21—H21C	109.5
C13—C18—H18	119.3	H21B—C21—H21C	109.5
			
C10—N2—C7—C4	113.63 (10)	C14—C13—C18—C17	1.38 (17)
C10—N2—C7—C8	−7.91 (11)	C8—C13—C18—C17	−179.57 (10)
O1—N1—C1—C2	−7.48 (17)	C6—C1—C2—C3	−0.44 (17)
O2—N1—C1—C2	173.19 (11)	N1—C1—C2—C3	178.89 (10)
O1—N1—C1—C6	171.88 (12)	C19—O5—C20—C21	−176.15 (10)
O2—N1—C1—C6	−7.46 (16)	C1—C2—C3—C4	−0.96 (17)
C2—C1—C6—C5	1.32 (17)	C2—C3—C4—C5	1.43 (17)
N1—C1—C6—C5	−178.01 (11)	C2—C3—C4—C7	−178.38 (10)
C20—O5—C19—O6	−3.51 (16)	N2—C7—C4—C3	−34.13 (14)
C20—O5—C19—C10	177.22 (9)	C8—C7—C4—C3	82.62 (13)
C22—O8—C23—C24	168.33 (11)	N2—C7—C4—C5	146.06 (10)
C7—N2—C10—C9	−18.33 (11)	C8—C7—C4—C5	−97.19 (12)
C7—N2—C10—C19	104.55 (10)	C18—C13—C14—C15	−0.44 (16)
C7—N2—C10—C22	−136.16 (9)	C8—C13—C14—C15	−179.43 (10)
C8—C9—C10—N2	37.62 (10)	C13—C18—C17—C16	−1.38 (18)
C8—C9—C10—C19	−82.98 (10)	C18—C17—C16—C15	0.42 (18)
C8—C9—C10—C22	153.23 (9)	C12—O4—C11—O3	5.05 (17)
O6—C19—C10—N2	12.31 (15)	C12—O4—C11—C8	−174.76 (10)
O5—C19—C10—N2	−168.42 (9)	C9—C8—C11—O3	−2.64 (16)
O6—C19—C10—C9	129.15 (11)	C13—C8—C11—O3	118.22 (12)
O5—C19—C10—C9	−51.58 (12)	C7—C8—C11—O3	−114.27 (12)
O6—C19—C10—C22	−106.11 (12)	C9—C8—C11—O4	177.16 (9)
O5—C19—C10—C22	73.16 (11)	C13—C8—C11—O4	−61.97 (11)
C10—C9—C8—C11	−159.73 (9)	C7—C8—C11—O4	65.53 (12)
C10—C9—C8—C13	81.23 (10)	C17—C16—C15—C14	0.51 (19)
C10—C9—C8—C7	−41.32 (10)	C13—C14—C15—C16	−0.49 (18)
C14—C13—C8—C11	110.59 (11)	C23—O8—C22—O7	−0.31 (18)
C18—C13—C8—C11	−68.40 (12)	C23—O8—C22—C10	177.58 (10)
C14—C13—C8—C9	−128.00 (11)	N2—C10—C22—O7	83.83 (14)
C18—C13—C8—C9	53.01 (12)	C9—C10—C22—O7	−29.43 (16)
C14—C13—C8—C7	−14.60 (15)	C19—C10—C22—O7	−155.97 (11)
C18—C13—C8—C7	166.41 (10)	N2—C10—C22—O8	−94.08 (11)
N2—C7—C8—C11	148.71 (9)	C9—C10—C22—O8	152.66 (9)
C4—C7—C8—C11	28.62 (12)	C19—C10—C22—O8	26.12 (12)
N2—C7—C8—C9	30.54 (10)	C1—C6—C5—C4	−0.82 (18)
C4—C7—C8—C9	−89.54 (10)	C3—C4—C5—C6	−0.51 (17)
N2—C7—C8—C13	−88.36 (10)	C7—C4—C5—C6	179.30 (10)
C4—C7—C8—C13	151.56 (9)		

### Hydrogen-bond geometry (Å, °)

**Table:**

*D*—H···*A*	*D*—H	H···*A*	*D*···*A*	*D*—H···*A*
N2—H1···O7^i^	0.88 (1)	2.59 (1)	3.360 (4)	148.(1)
C12—H12C···O1^ii^	0.96	2.58	3.360 (2)	139.(1)

Symmetry codes: (i) −*x*+1, *y*+1/2, −*z*+3/2; (ii) *x*+1, *y*, *z*.
